# Motion compensated reconstruction from free breathing 2D radial cardiac MRI data

**DOI:** 10.1186/1532-429X-18-S1-O110

**Published:** 2016-01-27

**Authors:** André Fischer, Anne Menini, Aurelien Bustin, Kevin M Johnson, Christopher J Francois, Anja C Brau

**Affiliations:** 1 Cardiac Center of Excellence, GE Healthcare, Garching, Germany; 2 grid.14003.360000000099041312Medical Physics, University of Wisconsin, Madison, WI USA; 3 grid.14003.360000000099041312Radiology, University of Wisconsin, Madison, WI USA; 4 grid.418145.dGE Global Research, Garching, Germany; 5 grid.6936.a0000000123222966Computer Science, Technische Universität München, Munich, Germany

## Background

Cardiac magnetic resonance imaging (CMR) is affected by both cardiac and respiratory motion. While ECG-gated imaging within a breath hold is often the method of choice to limit motion-related artifacts, free-breathing methods are favorable in patients with limited breath hold capability. Free-breathing approaches require either rapid single-shot scans to reduce respiratory motion artifacts at the expense of spatial resolution or higher resolution segmented respiratory-gated scans at the expense of scan time efficiency. Previous work has exploited the favorable properties (e.g., motion robustness, uniform sampling density) of Golden Angle [1] radial sampling including motion. Recently introduced motion compensated reconstructions [2,3] have been applied to various clinical applications. In this work, we propose to combine a 2D radial Golden Angle data acquisition scheme with a recently developed motion compensated reconstruction strategy [4] to obtain high-resolution motion compensated CMR data from time-efficient cardiac-gated free-breathing exams.

## Methods

**Figure 1:**
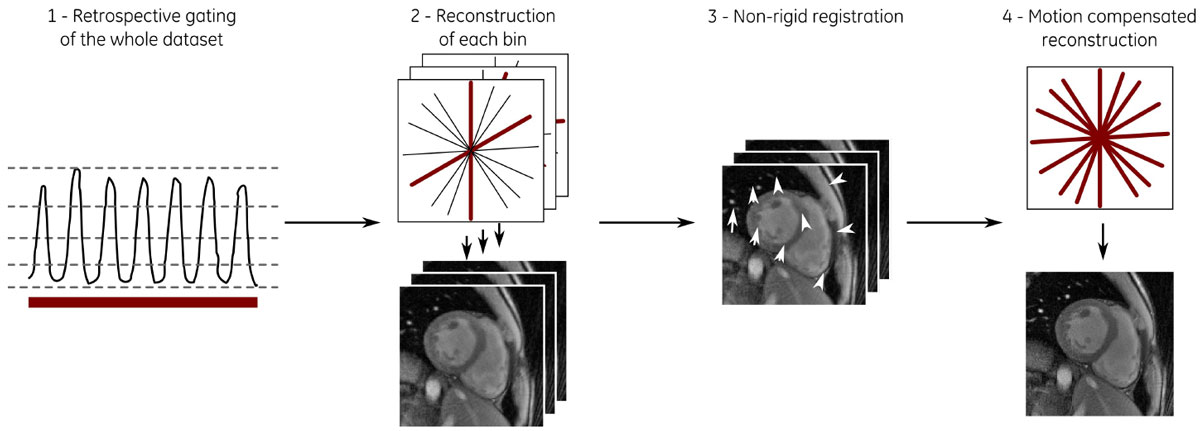
**Scheme of the motion compensated reconstruction (MCR)**.

A cardiac gated 2D golden angle radial spoiled gradient echo sequence with the following parameters was used: α = 15°, BW = ± 125 kHz, 256 readout points, TR = 4.26 ms, TE = 1.50 ms, FOV = 360 × 360 mm^2^. Data were acquired in diastole, total scan time was 50s. The respiratory belt signal was recorded synchronously with the MR acquisition. Three images were reconstructed from free-breathing data corresponding to 50s, 6s, and to 3s using a non-Cartesian iterative SENSE reconstruction [5]: 1) Combining all free-breathing data without motion management (FB), 2) Retrospective gating (respiratory belt signal) using data closest to end-expiration (RG), 3) Motion Compensated Reconstruction (MCR). The MCR is obtained in 4 steps: a) clustering of the all data into 6 respiratory bins according to the respiratory belt signal, b) independent reconstruction of the 6 bins, c) extraction of the motion between the bins by applying a non-rigid registration, c) model based reconstruction utilizing a motion-compensated SENSE-like reconstruction [6,7] (see also Figure 1).

## Results

**Figure 2:**
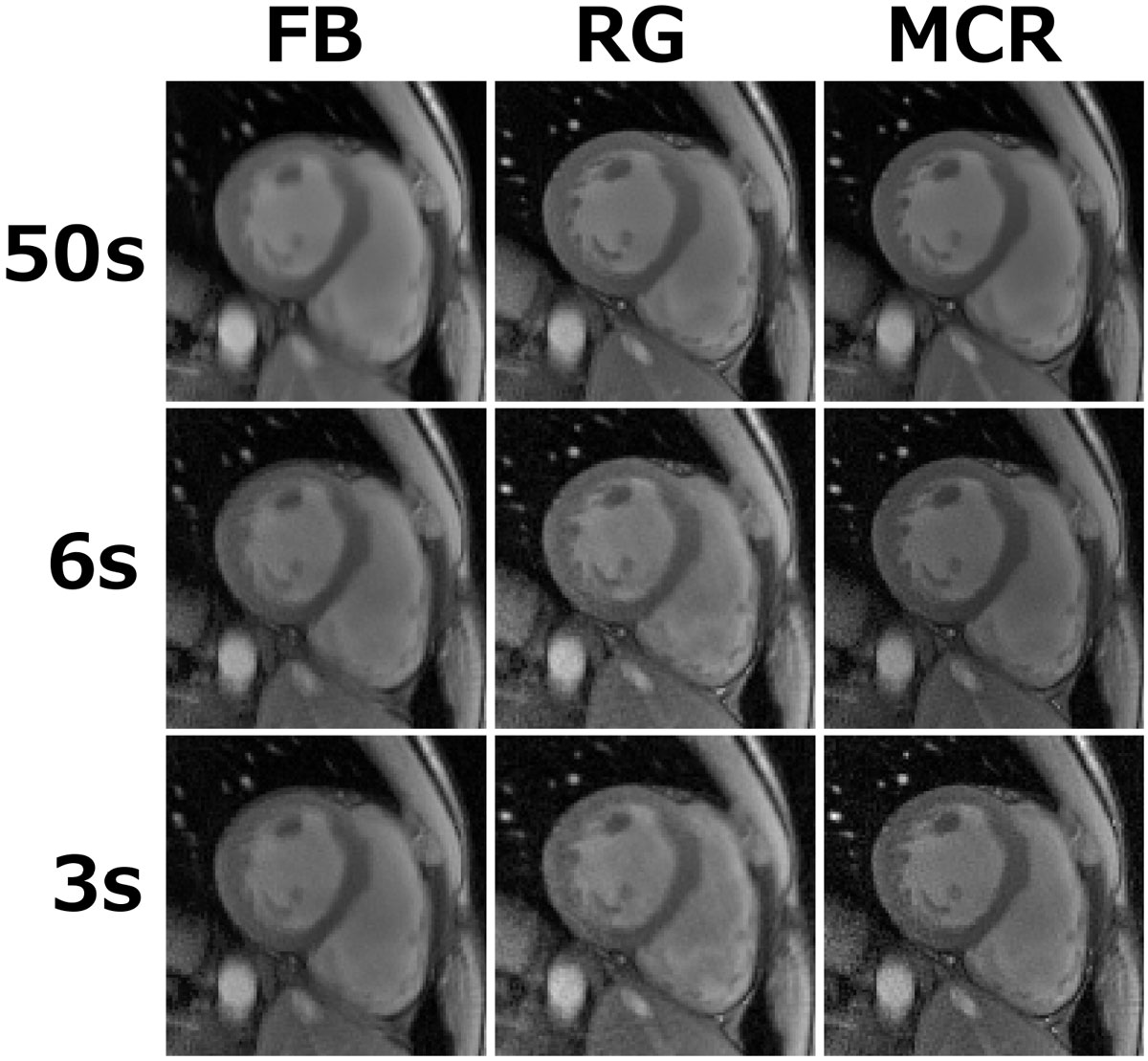
**Comparison of reconstruction results**.

Figure 2 compares reconstruction strategies. Combining all FB data into one dataset leads to significant blurring. RG improves apparent sharpness; however, small anatomical structures are best visualized in the MCR. The benefit of the MCR is more pronounced in the 6s and 3s examples.

## Conclusions

Motion compensated reconstruction is a promising technique to remove respiratory motion. By combining multiple respiratory bins into one spatially high-resolved, motion-corrected dataset, images with high visual sharpness are obtained in a time-efficient manner. Cardiac clinical applications such as function, perfusion, or late enhancement can potentially benefit from the presented data acquisition and reconstruction strategy and are are currently under investigation.

